# Supplementary material for: Combined lactate- and phosphate-dependent cytoplasmic acidification drives Mycobacterium tuberculosis growth arrest at acidic pH
Source: bioRxiv. 2026 May 16:2026.05.15.725484. Preprint. [Version 1] doi: 10.64898/2026.05.15.725484 (PMC13192937; doi:10.64898/2026.05.15.725484)
Supplement: Supplement 2 [file NIHPP2026.05.15.725484v1-supplement-2.pdf]

bioRxiv preprint doi: https://doi.org/10.64898/2026.05.15.725484; this version posted May 16, 2026. The copyright holder for this preprint (which was not certified by peer review) is the author/funder, who has granted bioRxiv a license to display the preprint in perpetuity. It is made available under a CC-BY 4.0 International license.

Combined lactate- and phosphate-dependent cytoplasmic acidification drives *Mycobacterium tuberculosis* growth arrest at acidic pH

Adam P. Kibiloski[1], Shelby J. Dechow[1], Bassel J. Abdalla[1], Heather M. Murdoch[1], Anna D. Tischler[2], Robert B. Abramovitch[1*]

Supplemental Figures

Figure S1. Growth arrest on lactate can be overcome through pyruvate supplementation or genetic mutation.

A) WT CDC1551 was grown in minimal medium buffered to pH 5.7 (red) and 7.0 (blue) supplemented with 4 mM lactate. On day 9, 10 mM pyruvate was added to Mtb grown on lactate at pH 5.7 (green). This experiment was performed once in biological duplicates.

B) Growth curves of the transposon mutants grown on 4 mM lactate at acidic pH 5.7 over 21 days, OD_{600} were measured on day 9, day 13 and day 21. This experiment was performed once in biological duplicate

Figure S2. Growth arrest in lactate can be overcome in both WT H37Rv and WT Erdman by depleting phosphate.

Growth of WT Erdman strain and WT H37Rv in medium supplemented with 4 mM lactate at pH 5.7 in medium either containing phosphate (purple) or not containing phosphate (blue) after 21 days. Dashed line indicates growth arrest phenotype. Statistical analysis was performed using an unpaired t-test (***, $p<0.001$). Experiment was performed twice. Error bars represent the standard deviation of three technical replicates.

Figure S3. Mtb growth arrest on lactate is dependent on phosphate concentration.

Mtb grown at varying lactate concentrations in media amended with (A) 80 µM, (B) 32 µM, (C) 2.05 µM, and (D) 0.8 µM phosphate. Statistical analyses were performed using an unpaired t-test (*, $p<0.05$ **, $p<0.005$ ***, $p<0.001$). The experiment was repeated twice with similar results. The error bars represent the standard deviation of three technical replicates. This experiment was performed at least twice. Error bars represent the standard deviation of three technical triplicates.

Figure S4. Growth arrest on lactate is dependent on phosphate concentration.

Mtb grown at varying lactate concentrations in media amended with (A) 40 mM, (B) 16 mM, (C) 6.4 mM, (D) 2.56 mM, (E) 1.024 mM, (F) 0.41 mM lactate. Statistical analyses were performed using an unpaired t-test (***, $p<0.001$). This experiment was performed at least twice. Error bars represent the standard deviation of three technical triplicates.

bioRxiv preprint doi: https://doi.org/10.64898/2026.05.15.725484; this version posted May 16, 2026. The copyright holder for this preprint (which was not certified by peer review) is the author/funder, who has granted bioRxiv a license to display the preprint in perpetuity. It is made available under a CC-BY 4.0 International license.

Figure S5. Growth arrest on lactate is pH dependent.

A) Growth of WT (purple), *phoT::Tn* (light blue), and the complement (teal) was evaluated after 21 days. Statistical significance was evaluated using an unpaired t-test (***, $p<0.001$). This experiment was performed once. Error bars represent the standard deviation of three technical replicates.

B) Cytoplasmic pH measurements of Mtb exposed to either pyruvate (purple) or glycerol (teal). This experiment was performed at least twice with similar results. Error bars represent the standard deviation of three technical replicates.

C) Endpoint data from the cytoplasmic pH time course experiment where. All cytoplasmic pHs' were compared to the WT DMSO control exposed to no lactate or phosphate. Statistical significance was evaluated using an unpaired t-test (*, $p<0.05$). This experiment was performed twice with similar results. Error bars represent the standard deviation of three technical replicates.

Figure S6. Cytoplasmic pH in not impacted in strains exposed to lactate and phosphate at neutral pH.
WT*, phoT::Tn,* and complemented strains exposed to a range of concentrations of lactate and phosphate at neutral pH. Experiment were performed at least twice with similar results. Error bars represent the standard deviation of three technical triplicates.

Figure S7. Lactate and phosphate at low concentrations have modest impacts on membrane potential.

A) WT, *phoT::Tn*, and complemented strains membrane potential monitored over time at pH 5.7 with endpoint data plotted. Statistical significance determined by unpaired t-test (***, $p<0.001$).

B) WT, *phoT::Tn*, and complemented strains membrane potential monitored over time at pH 7.0 with endpoint data plotted. Statistical significance determined by unpaired t-test (*, $p<0.05$, **, $p<0.005$, ***, $p<0.001$). Both experiments were performed at least twice with similar results. Error bars represent the standard deviation of three technical triplicates.

Figure S8. The *phoT::Tn* mutant has higher membrane potential in pyruvate and glycerol.
Membrane potential of strains incubated in MMAT medium with high phosphate (25 mm) with different carbon sources. The strains were evaluated were *phoT::Tn* (pVV16::p*hoT*) (teal), *phoT::Tn* (light blue), and WT (purple). WT treated with CCCP (orange) was used as a depolarized control. This experiment was performed twice with similar results. Error bars represent the standard deviation of three technical replicates.

Figure S9. Dose-response curves of Electron transport chain-targeting agents across different growth conditions and genetic backgrounds. ETC-targeting agents were tested against WT, *phoT::Tn*, and *phoT::Tn* (pMV306::*phoT*) strains grown in different carbon sources (Lactate and Pyruvate), pHs (6 and 7), and in the presence and absence of phosphate to demonstrate the role of the lactate-induced ETC stress in potentiating the efficacy of ETC-targeting agents. TDZ: Thioridazine, CCCP: Carbonyl cyanide m-chlorophenyl hydrazone, VAL: Valinomycin, BDQ: Bedaquiline, CLZ: Clofazimine, NIG: Nigericin.

bioRxiv preprint doi: https://doi.org/10.64898/2026.05.15.725484; this version posted May 16, 2026. The copyright holder for this preprint (which was not certified by peer review) is the author/funder, who has granted bioRxiv a license to display the preprint in perpetuity. It is made available under a CC-BY 4.0 International license.

Figure S10. Genes differentially regulated when comparing pH 5.7 to 7.0 in the *phoT::*Tn mutant or WT Mtb.

A) Heat map of genes differentially regulated (fold-change>1.5, q<0.05) in the *phoT::Tn* mutant at pH 5.7 compared to the *phoT::Tn* mutant at pH 7.0 (left) and WT grown at pH 5.7 compared to WT grown at pH 7.0 (right).
B) Magnitude/amplitude plots of average Log_2 counts per million (CPM) and Log_2 fold-change of the *phoT::Tn* mutant grown at pH 5.7 compared to the *phoT::Tn* mutant grown at pH 7.0.
C) Magnitude/amplitude plots of average Log_2 counts per million (CPM) and Log_2 fold-change of WT grown at pH 5.7 compared to WT grown at pH 7.0.
D) Venn diagram depicting the overlap of genes upregulated in both strains at pH 5.7 compared to pH 7.0. Overlapping region indicates genes upregulated/downregulated in both strains at pH 5.7. Blue region indicates genes only upregulated in the *phoT::Tn* mutant at pH 5.7. The yellow region indicates genes only upregulated in WT at pH 5.7.

Figure S11. Δ*phoT* mutant validation.

A) Diagram of the *phoT* locus. Black Δ*phoT* bar indicates the region deleted. Δ*phoT*-F and Δ*phoT*-R (red) are primers used to confirm the Δ*phoT* deletion by PCR. XhoI restriction enzyme sites and probe (purple) used for Southern blotting are also shown.
B) PCR confirmation of the Δ*phoT* mutation using primers Δ*phoT*-F and Δ*phoT*-R on genomic DNA from WT and Δ*phoT*. Sizes of the DNA ladder in kb are indicated.
C) Southern blotting confirmation of the Δ*phoT* mutation. Genomic DNA from WT and Δ*phoT* mutant *Mtb* was digested with XhoI, separated on a 0.8% TAE gel and transferred to a Hybond N+ nylon membrane (Amersham). A Δ*phoT* probe generated by PCR was labeled with the ECL direct nucleic acid labeling kit (Amersham). The blot was incubated with the probe overnight in Amersham Gold hybridization buffer with 0.5M NaCl and 5% blocking reagent at 42˚C. Bands that hybridized to the probe were detected with ECL detection reagents (Amersham), autoradiographic film and an automated film processor. Positions of molecular size markers in kb are indicated. Δ*phoT* removes an XhoI site, so the expected size of the band is larger, ~4.2 kb, as compared to ~3.0 kb for WT.
D) Phthiocerol dimycocerosate (PDIM) was detected in ^{14}C propionate labeled apolar lipid extracts from WT and Δ*phoT* by thin layer chromatography. Spots corresponding to phthiocerol (methoxy; DIM A) and phthiodiolone (keto; DIM B) forms are indicated.

Figure S12. *phoT* mutants grow on glycerol, glucose, and lactate at acidic pH on modified 7H9 medium.

A) Δ*phoT*, *phoT::Tn* mutant, WT and complemented strain growing at pH 5.0 on 0.2% (v/v) glycerol in carbon depleted 7H9.
B) Δ*phoT*, *phoT::Tn* mutant, WT and complemented strain growth arrested on 2% (w/v) glucose at pH 5.0 in carbon depleted 7H9.
C) Carbon panel of WT Erdman grown in MMAT at pH 5.7 in both phosphate rich medium, as well as phosphate replete medium. All three experiments were performed at least twice with similar results. Error bars represent the standard deviation of three technical replicates.

bioRxiv preprint doi: https://doi.org/10.64898/2026.05.15.725484; this version posted May 16, 2026. The copyright holder for this preprint (which was not certified by peer review) is the author/funder, who has granted bioRxiv a license to display the preprint in perpetuity. It is made available under a CC-BY 4.0 International license.

Figure S13. Subset of downregulated and upregulated genes are specific to lactate at acidic pH.
Venn diagram depicting the overlay of genes upregulated in WT in either lactate, pyruvate, or glycerol at pH 5.7 compared to WT grown in the same carbon source at pH 7.0. The center depicts genes upregulated at acidic pH. The top left depicts genes upregulated only in lactate. The top right depicts genes only upregulated in glycerol. The bottom indicates genes only upregulated in pyruvate. In the venn diagram on the right, the center depicts genes downregulated at acidic pH in all conditions. The top left depicts genes downregulated only in lactate. The top right depicts genes only downregulated in glycerol. The bottom indicates genes only downregulated in pyruvate.

bioRxiv preprint doi: https://doi.org/10.64898/2026.05.15.725484; this version posted May 16, 2026. The copyright holder for this preprint (which was not certified by peer review) is the author/funder, who has granted bioRxiv a license to display the preprint in perpetuity. It is made available under a CC-BY 4.0 International license.

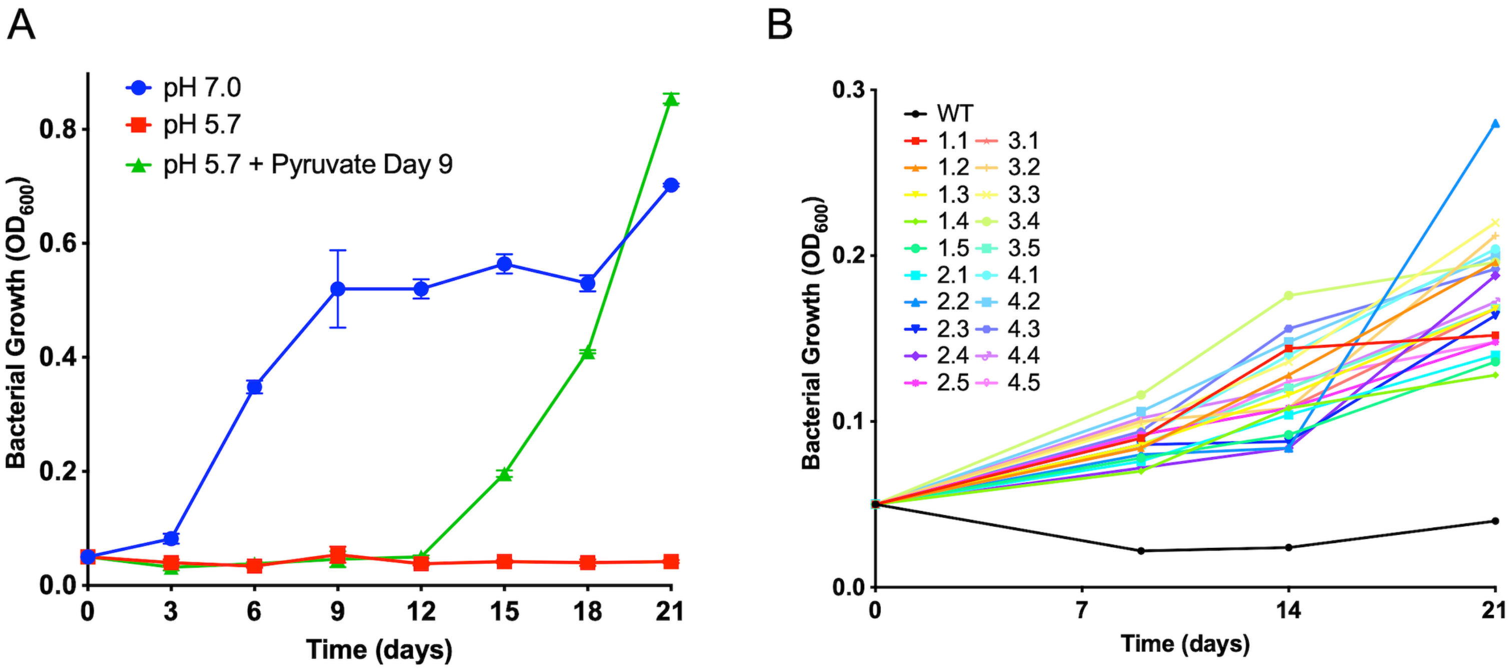

Supplemental Figure 1

bioRxiv preprint doi: https://doi.org/10.64898/2026.05.15.725484; this version posted May 16, 2026. The copyright holder for this preprint (which was not certified by peer review) is the author/funder, who has granted bioRxiv a license to display the preprint in perpetuity. It is made available under a CC-BY 4.0 International license.

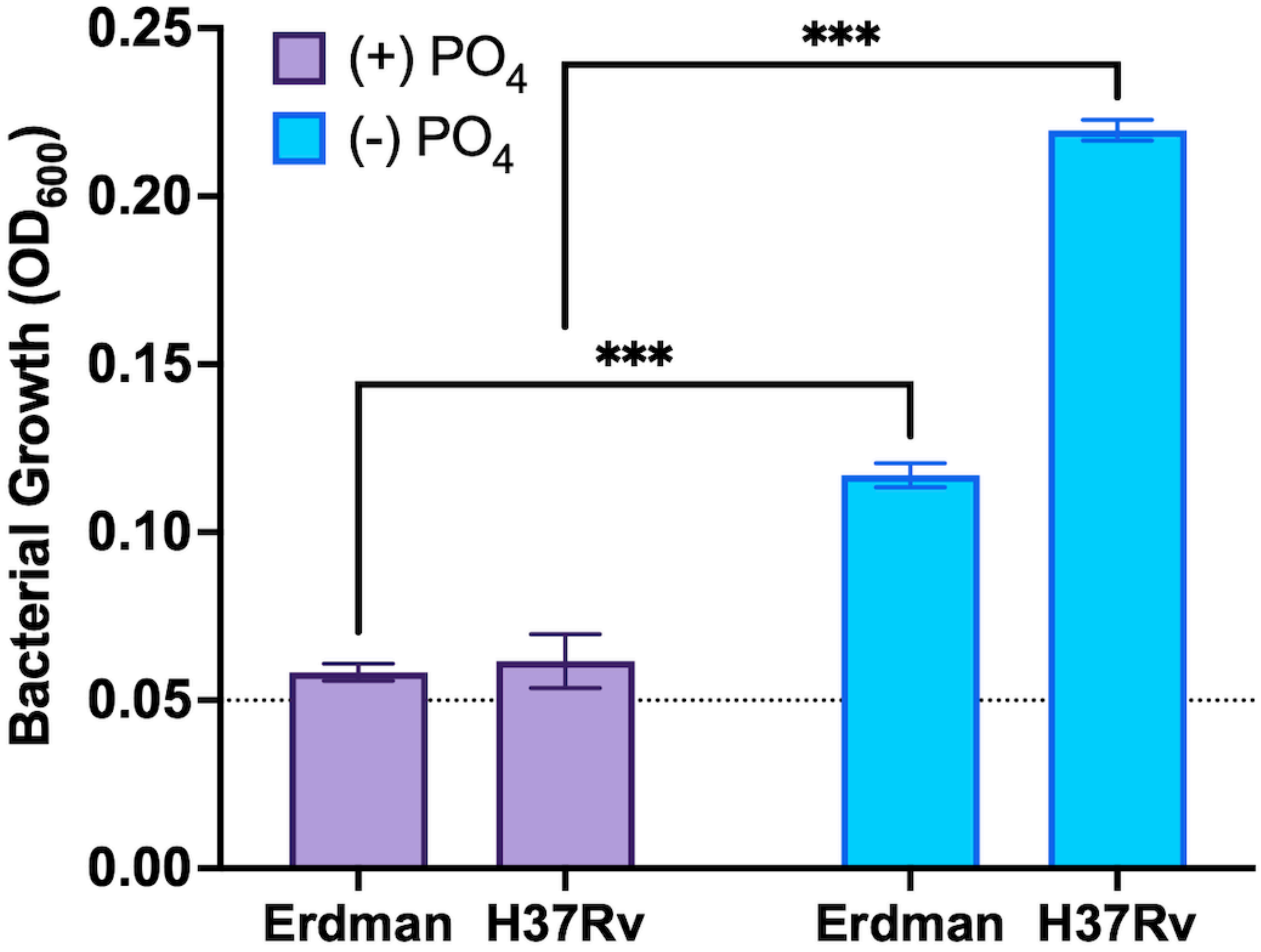

Supplemental Figure 2

bioRxiv preprint doi: https://doi.org/10.64898/2026.05.15.725484; this version posted May 16, 2026. The copyright holder for this preprint (which was not certified by peer review) is the author/funder, who has granted bioRxiv a license to display the preprint in perpetuity. It is made available under a CC-BY 4.0 International license.

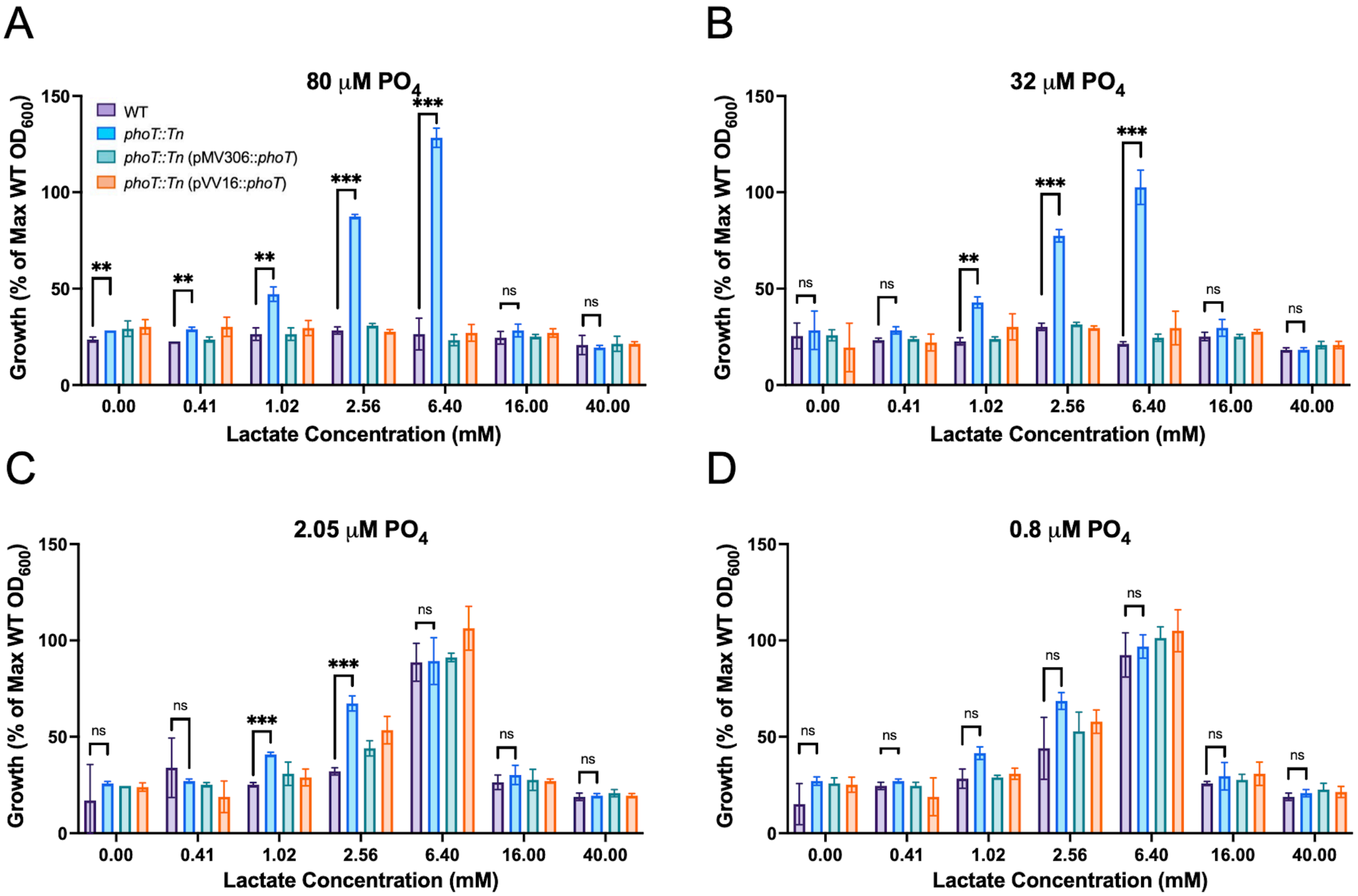

Supplemental Figure 3

bioRxiv preprint doi: https://doi.org/10.64898/2026.05.15.725484; this version posted May 16, 2026. The copyright holder for this preprint (which was not certified by peer review) is the author/funder, who has granted bioRxiv a license to display the preprint in perpetuity. It is made available under a CC-BY 4.0 International license.

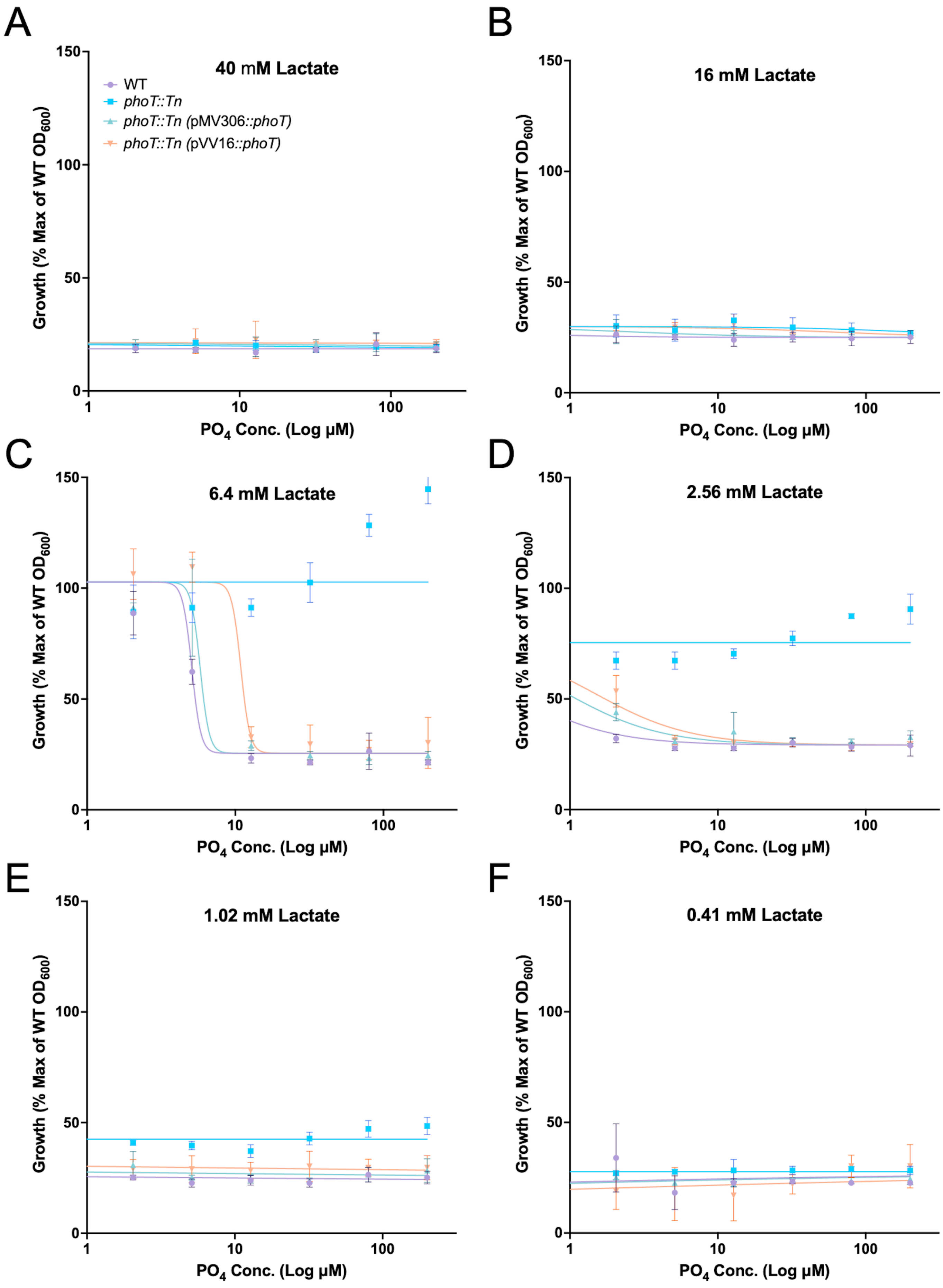

Supplemental Figure 4

bioRxiv preprint doi: https://doi.org/10.64898/2026.05.15.725484; this version posted May 16, 2026. The copyright holder for this preprint (which was not certified by peer review) is the author/funder, who has granted bioRxiv a license to display the preprint in perpetuity. It is made available under a CC-BY 4.0 International license.

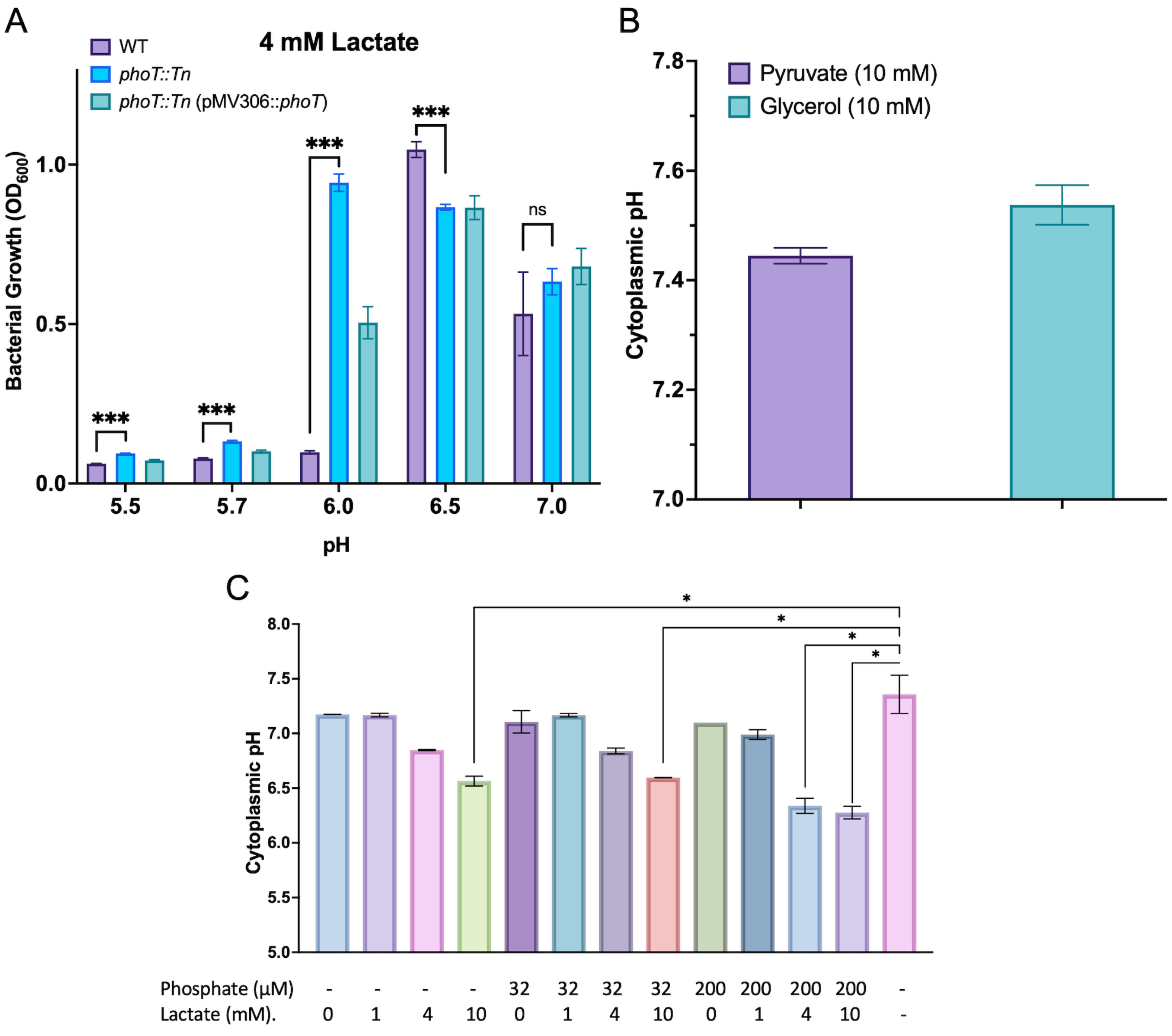

Supplemental Figure 5

bioRxiv preprint doi: https://doi.org/10.64898/2026.05.15.725484; this version posted May 16, 2026. The copyright holder for this preprint (which was not certified by peer review) is the author/funder, who has granted bioRxiv a license to display the preprint in perpetuity. It is made available under a CC-BY 4.0 International license.

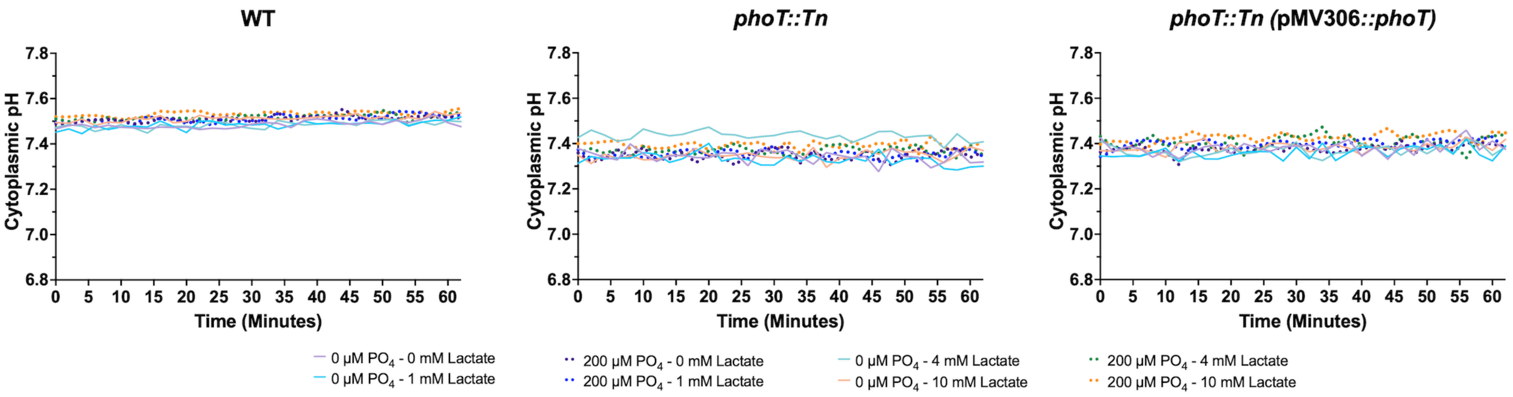

Supplemental Figure 6

bioRxiv preprint doi: https://doi.org/10.64898/2026.05.15.725484; this version posted May 16, 2026. The copyright holder for this preprint (which was not certified by peer review) is the author/funder, who has granted bioRxiv a license to display the preprint in perpetuity. It is made available under a CC-BY 4.0 International license.

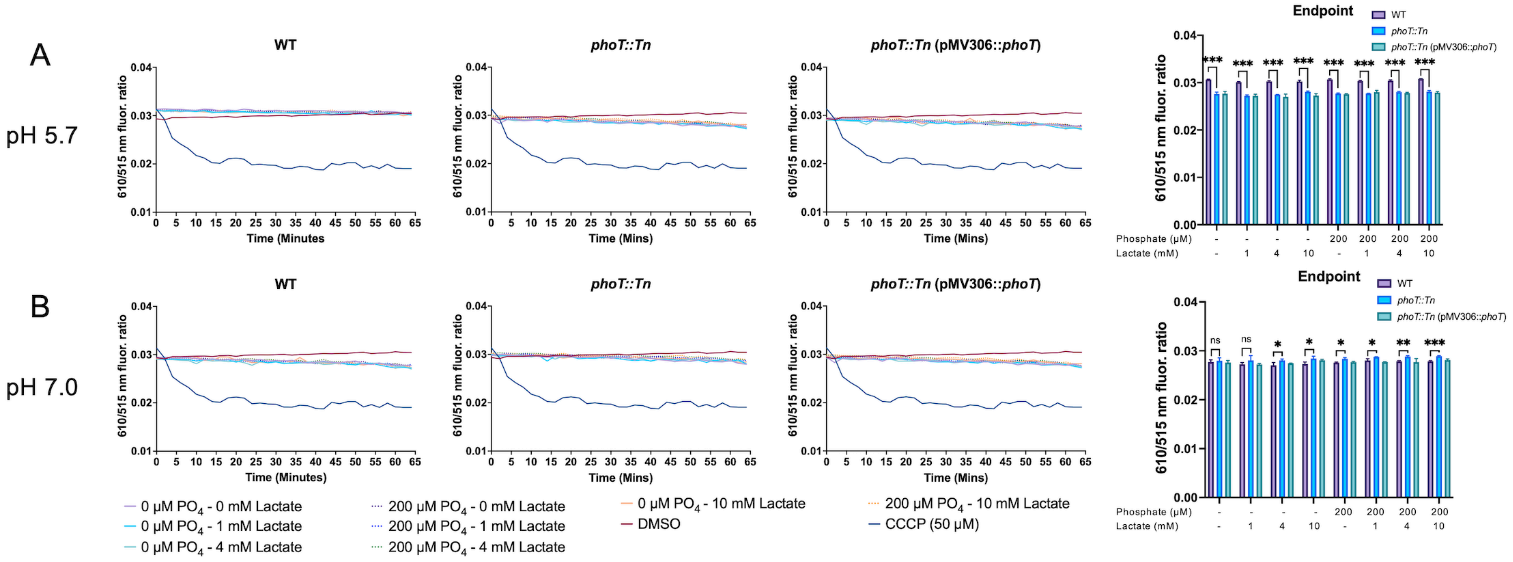

Supplemental Figure 7

bioRxiv preprint doi: https://doi.org/10.64898/2026.05.15.725484; this version posted May 16, 2026. The copyright holder for this preprint (which was not certified by peer review) is the author/funder, who has granted bioRxiv a license to display the preprint in perpetuity. It is made available under a CC-BY 4.0 International license.

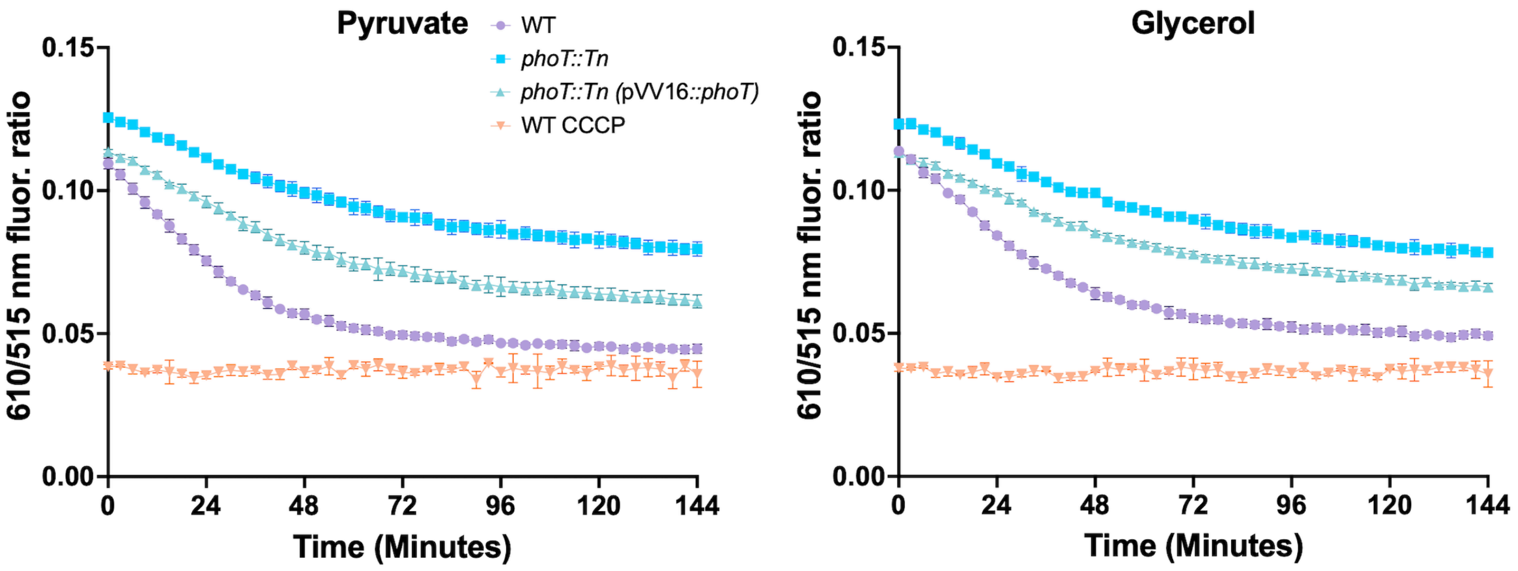

Supplemental Figure 8

bioRxiv preprint doi: https://doi.org/10.64898/2026.05.15.725484; this version posted May 16, 2026. The copyright holder for this preprint (which was not certified by peer review) is the author/funder, who has granted bioRxiv a license to display the preprint in perpetuity. It is made available under a CC-BY 4.0 International license.

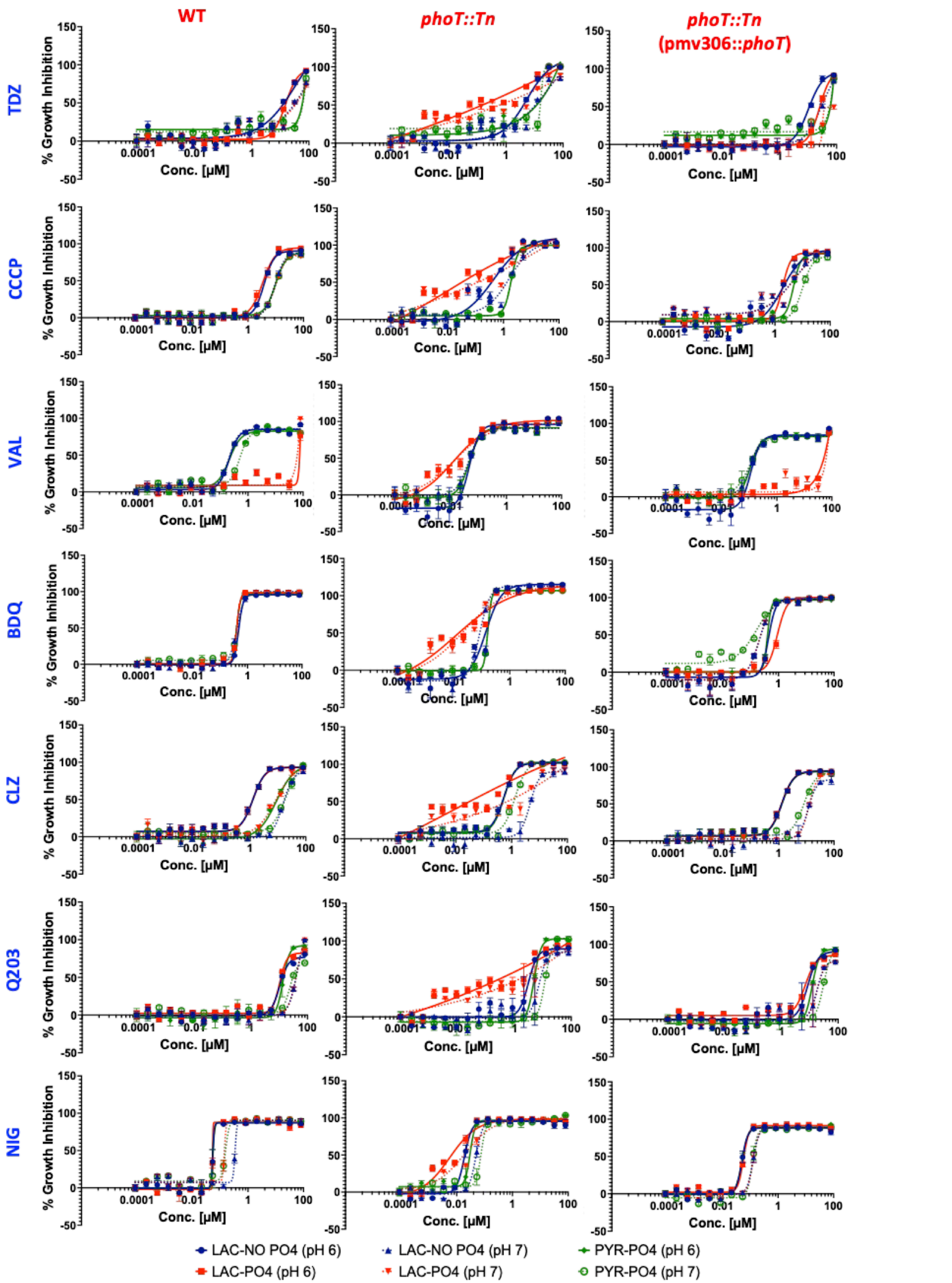

Supplemental Figure 9

bioRxiv preprint doi: https://doi.org/10.64898/2026.05.15.725484; this version posted May 16, 2026. The copyright holder for this preprint (which was not certified by peer review) is the author/funder, who has granted bioRxiv a license to display the preprint in perpetuity. It is made available under a CC-BY 4.0 International license.

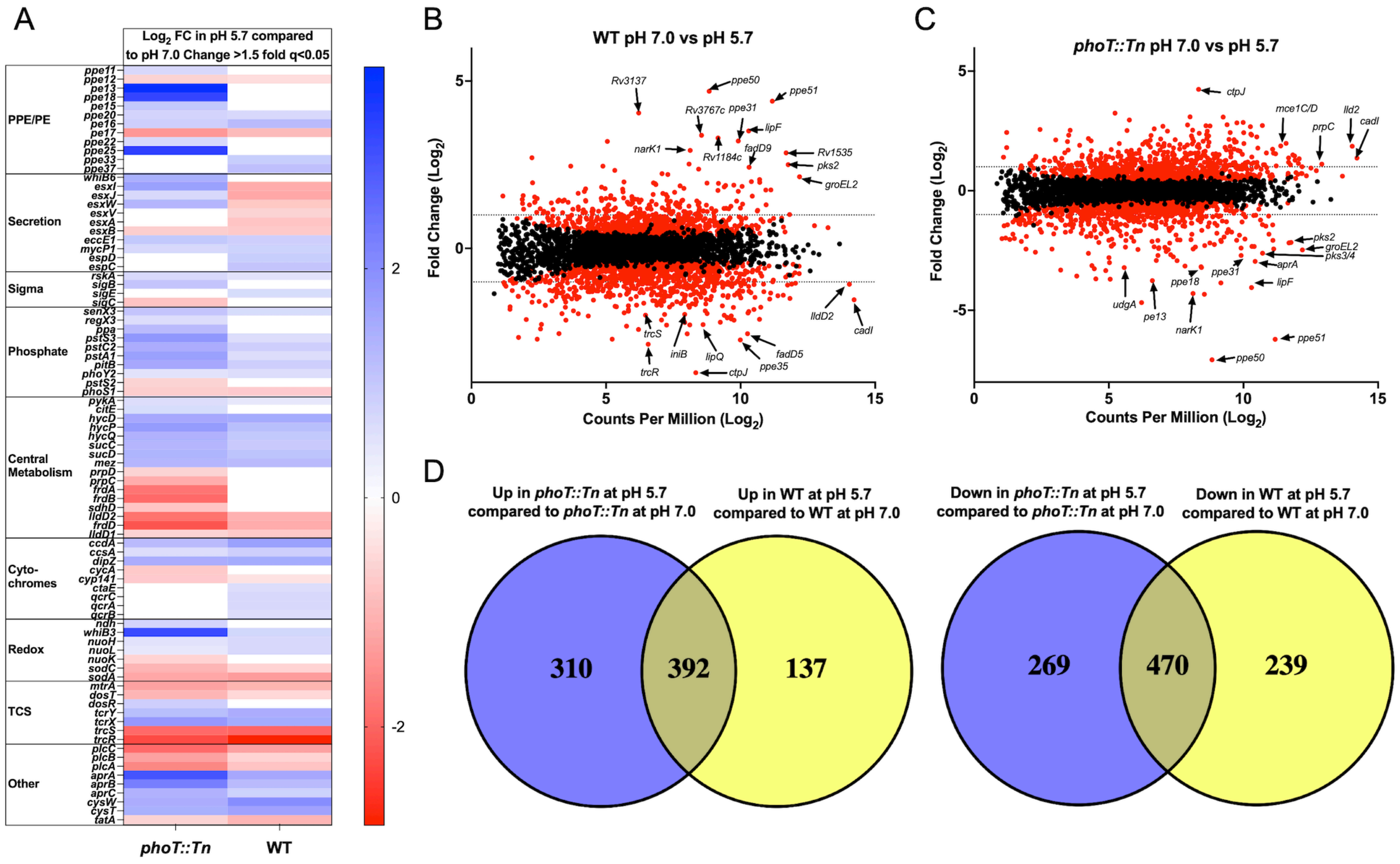

Supplemental Figure 10

bioRxiv preprint doi: https://doi.org/10.64898/2026.05.15.725484; this version posted May 16, 2026. The copyright holder for this preprint (which was not certified by peer review) is the author/funder, who has granted bioRxiv a license to display the preprint in perpetuity. It is made available under a CC-BY 4.0 International license.

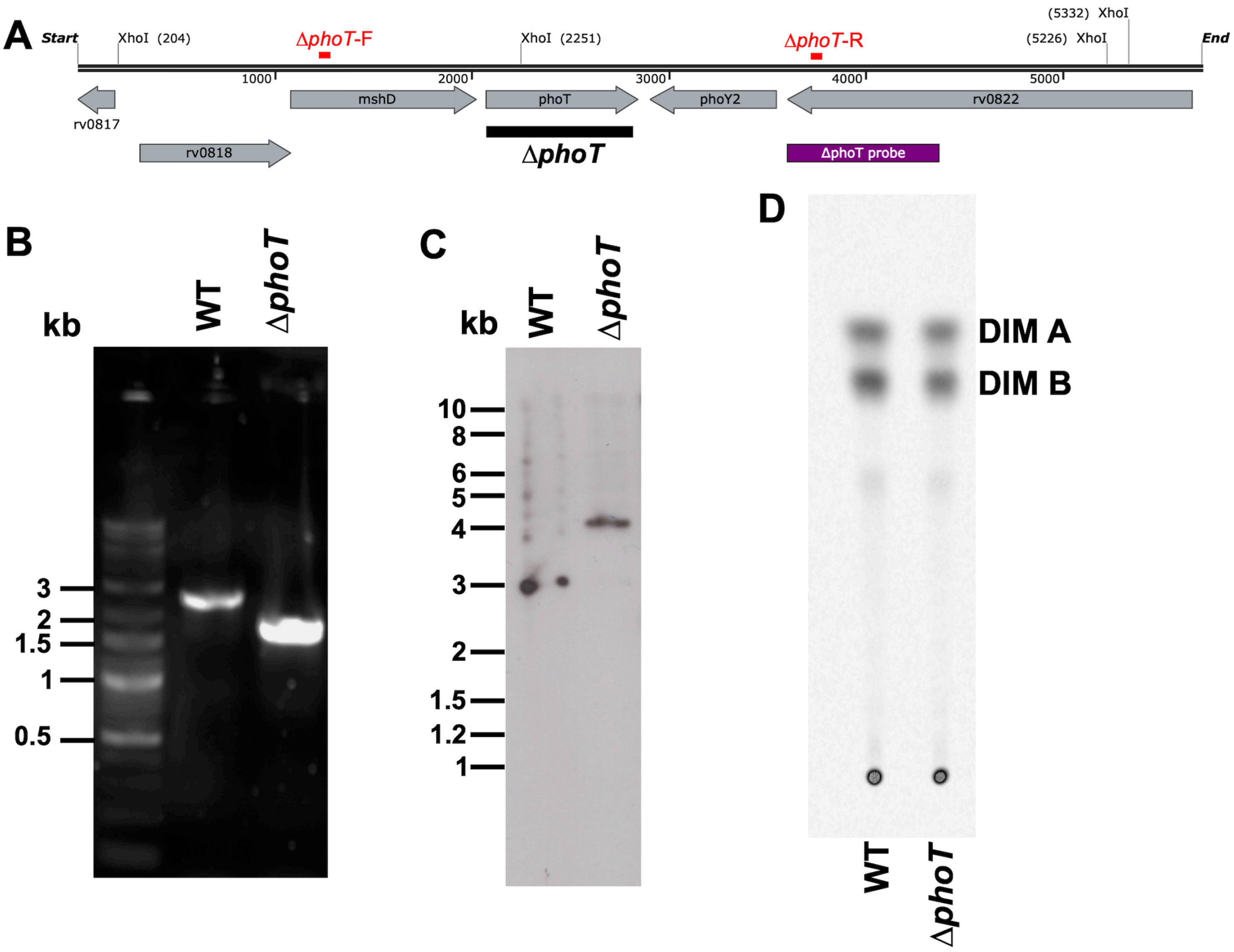

Supplemental Figure 11

bioRxiv preprint doi: https://doi.org/10.64898/2026.05.15.725484; this version posted May 16, 2026. The copyright holder for this preprint (which was not certified by peer review) is the author/funder, who has granted bioRxiv a license to display the preprint in perpetuity. It is made available under a CC-BY 4.0 International license.

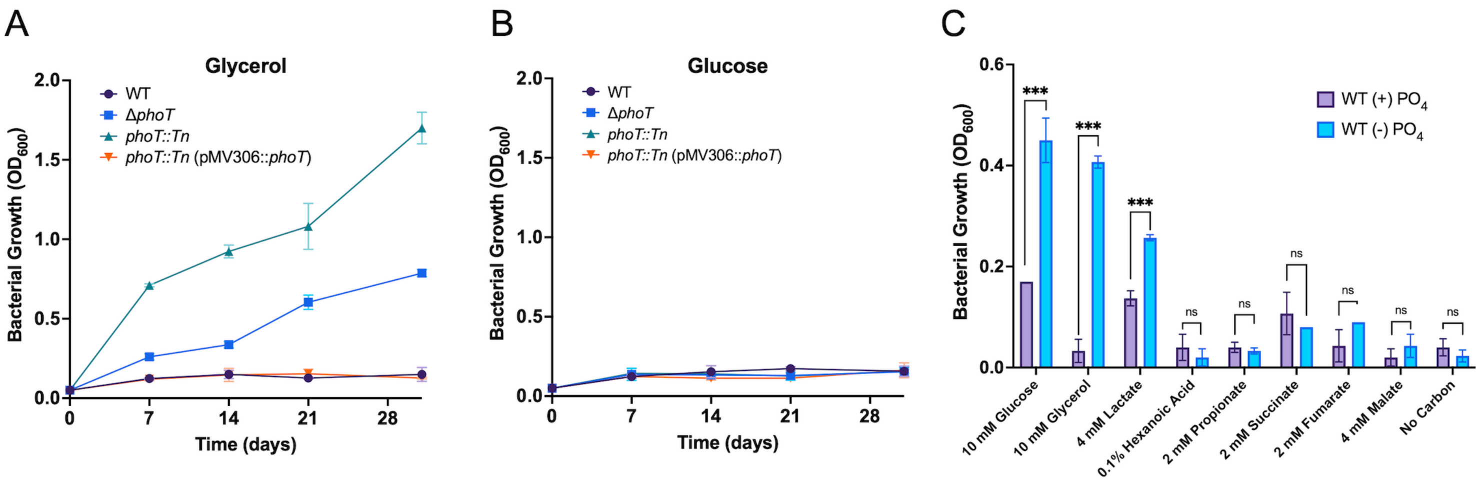

Supplemental Figure 12

bioRxiv preprint doi: https://doi.org/10.64898/2026.05.15.725484; this version posted May 16, 2026. The copyright holder for this preprint (which was not certified by peer review) is the author/funder, who has granted bioRxiv a license to display the preprint in perpetuity. It is made available under a CC-BY 4.0 International license.

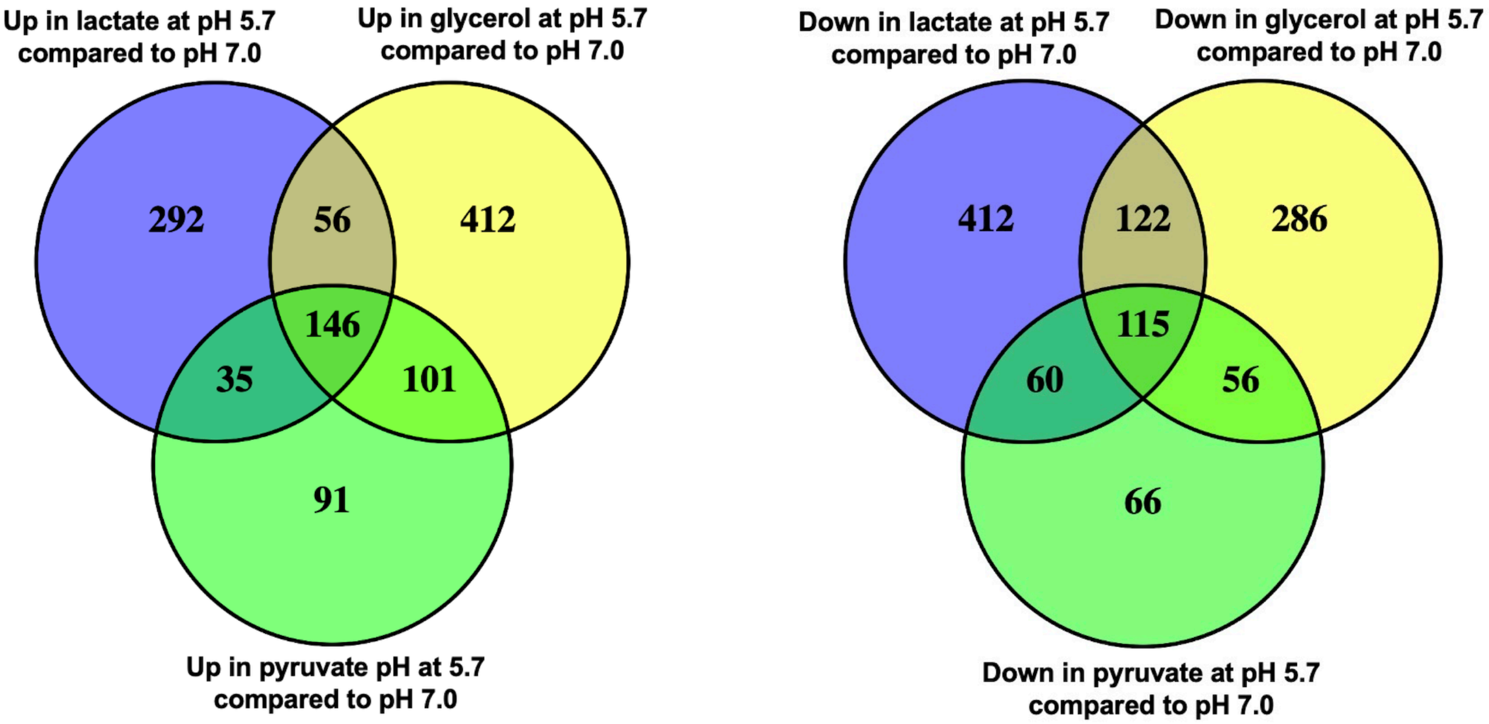

Supplemental Figure 13
